# A critical assessment of the association between postnatal toxoplasmosis and epilepsy in immune-competent patients

**DOI:** 10.1007/s10096-016-2897-0

**Published:** 2017-01-12

**Authors:** J. W. Uzorka, S. M. Arend

**Affiliations:** 1 Department of Infectious Diseases, C5P-40, Leiden University Medical Center, Albinusdreef 2, 2333 ZA Leiden, The Netherlands; 2 Faculty of Medicine, Leiden University, Leiden, The Netherlands

## Abstract

While postnatal toxoplasmosis in immune-competent patients is generally considered a self-limiting and mild illness, it has been associated with a variety of more severe clinical manifestations. The causal relation with some manifestations, e.g. myocarditis, has been microbiologically proven, but this is not unequivocally so for other reported associations, such as with epilepsy. We aimed to systematically assess causality between postnatal toxoplasmosis and epilepsy in immune-competent patients. A literature search was performed. The Bradford Hill criteria for causality were used to score selected articles for each component of causality. Using an arbitrary but defined scoring system, the maximal score was 15 points (13 for case reports). Of 704 articles, five case reports or series and five case–control studies were selected. The strongest evidence for a causal relation was provided by two case reports and one case–control study, with a maximal causality score of, respectively, 9/13, 10/13 and 10/15. The remaining studies had a median causality score of 7 (range 5–9). No selection bias was identified, but 6/10 studies contained potential confounders (it was unsure whether the infection was pre- or postnatal acquired, or immunodeficiency was not specifically excluded). Based on the evaluation of the available literature, although scanty and of limited quality, a causal relationship between postnatal toxoplasmosis and epilepsy seems possible. More definite proof requires further research, e.g. by performing *Toxoplasma* serology in all de novo epilepsy cases.

**Electronic supplementary material:**

The online version of this article (doi:10.1007/s10096-016-2897-0) contains supplementary material, which is available to authorized users.

## Introduction

Toxoplasmosis is caused by the intracellular parasite *Toxoplasma gondii*, which has felines as the definitive host and a variety of vertebrates as intermediate hosts. The clinical manifestations in humans depend on whether it is transmitted pre- or postnatally, on gestational age in case of prenatal transmission and on host immune status. A recent publication based on registered diagnoses from private insurance records reported significant morbidity and mortality related to toxoplasmosis, underscoring the relevance of the subject [1].

The seroprevalence of toxoplasmosis, as reflected by the presence of IgG antibodies against *T. gondii*, differs worldwide. In the USA, the seroprevalence was approximately 14% by the age of 40 years old [2], while in the Netherlands, the seroprevalence was 47% in the age category 40–44 years of age [3]. In postnatal infected immune-competent individuals, the clinical course is asymptomatic in 90% of the cases, while in the remaining 10%, it usually presents as lymphadenopathy, in which especially the cervical nodes are involved [4]. Lymphadenopathy can be accompanied by fever, rash, sore throat, hepatosplenomegaly and atypical lymphocytosis. Myocarditis, hepatitis, pneumonitis, polymyositis and encephalitis have been reported but are rare manifestations [4].

During the past several years, seropositivity for *T. gondii* has been associated with various diseases, including e.g. malignancies (e.g. breast cancer, melanoma or non-Hodgkin lymphoma) and auto-inflammatory diseases (systemic lupus erythematosus, rheumatoid arthritis or granulomatosis and polyangiitis) [5]. However, the association was neither supported by a solid hypothesis on the pathogenesis nor by evidence of causality and the actual role of *T. gondii* in these diseases therefore remains speculative. An association with neurological manifestations such as Alzheimer’s disease, Bell’s palsy, migraine and epilepsy has also been reported [5], which would be more plausible given the neurotropic nature of *T. gondii*. In a recently published meta-analysis by Ngoungou and colleagues, which included studies of immune-competent patients with toxoplasmosis as well as of patients with congenital toxoplasmosis or with human immunodeficiency virus (HIV) infection, it was concluded that toxoplasmosis should be regarded as a risk factor for epilepsy [6]. While the risk of epilepsy in congenital toxoplasmosis or in a setting of impaired immunity is unequivocal, this is not the case for postnatal toxoplasmosis in immune-competent individuals. In the present study we, therefore, specifically aimed to evaluate the possible causal role of postnatal toxoplasmosis in immune-competent patients for the development of epilepsy.

## Materials and methods

### Literature search

A literature search was performed on February 5th 2015 of the PubMed, Medline, Embase, Web of Science and Cochrane databases. The keywords were ‘toxoplasmosis’ and ‘epilepsy’; for the full search, see the supplementary material. An additional search was conducted on July 14th 2016 using Medline and the keywords were ‘toxoplasm* epilepsy 2015 2016’.

### Selection of studies

Articles were selected based on the title and, when potentially relevant, the abstract. Included were studies on toxoplasmosis and epilepsy published between 1966 and July 2016. Exclusion criteria were toxoplasmosis in patients with HIV infection or any other primary or secondary immune deficiency, congenital toxoplasmosis, publications in any language other than English, French or Dutch and animal studies.

### Scoring of causality (Bradford Hill)

In order to construct a semi-quantitative measure for causality in each article, the nine criteria for causality were used as published by Sir Austin Bradford Hill in 1965 [7]. These criteria consisted of strength, consistency, specificity, temporality, biological gradient, plausibility, coherence, experiment and analogy, as shown in Table 1, which shows the arbitrary scoring system that was specifically developed for this study. Scores for each article were given by both authors and in case of discrepancy, consensus was obtained. The maximal achievable score was 15 (for case reports, this was 13, as the criterion of strength was not applicable).

**Table 1:** Criteria and scoring method for assessment of the causality of postnatal toxoplasmosis for the development of epilepsy

Bradford Hill criteria	Explanation	Score (points)	Criteria for score
Strength	What was the strength of the association?	OR/RR is strong (2)	2: OR/RR ≥ 4 and significant
		OR/RR is weak (1)	1: OR/RR < 4 and significant
		No association (0)	0: No significant association
		Not applicable (NA)	NA: case report(s)
Consistency	Was the result found in different settings, by different authors?^a^	Yes (2)	2: if ≥ 2 different settings and ≥ 2 different groups
		Yes (1)	1: if ≥ 2 different settings or ≥ 2 different groups
		No (0)	0: only 1 setting and 1 group
Specificity	Was the tested group representative for a general conclusion?	Yes (2)	2: tested group reflects population of interest
		Partially (1)	1: tested group was randomly selected from a *subgroup* of the population of interest
		No (0)	0: strongly selected subjects
Temporality	Did the effect take place after the exposure?	Yes (2)	Note that exposure indicates infection with *T. gondii*
		No or not evaluable (0)	
Gradient	Is there a relation between the amount of exposure and (the severity of) the disease?	Yes (2)	For lack of alternative parameter, amount of exposure was defined as higher antibody titres to *T. gondii*
		No (0)	
Plausibility	Is the causation biologically plausible?^a^	Yes (1)	1: a plausible pathophysiological explanation is available
		No (0)	
Coherence	Is the relation between exposure and disease in conflict with our current data?^a^	No (1)	Current data consist of knowledge taught in standard medical textbooks or other sources (note that the lack of conflict yields a positive score).
		Yes (0)	
Experiment	Did anti-*Toxoplasma* treatment improve the alleged associated disease?	Yes (2)	2: treatment was randomised
		Yes (1)	1: treatment was not randomised
		No/not sure (0)	
Analogy	Are there similar associations?^a^	Yes (1)	1: any other infection associated with development of epilepsy
		No (0)	

^a^These four criteria were not applicable to individual articles but were scored simultaneously for all articles in the present study (see the Results section)

### Scoring for bias and confounding

Articles were qualitatively assessed on potential bias/confounding (comprised of information bias, selection bias and confounding).

### Citation index and citations

Articles were assessed with regard to scientific impact, using the impact factor at the time of publication and citation score at the time of the present study, as reported on the Web of Science.

## Results

The search performed in February 2015 resulted in 701 articles, of which nine fulfilled the selection criteria. 215 articles were excluded due an immune deficiency in the patients, 248 articles because it was primarily about a different subject, 63 were about congenital toxoplasmosis, 61 because of the language, 32 were animal studies, 72 were not accessible, while one was a meta-analysis of earlier studies [8]. The nine remaining articles consisted of three case reports, one case series and five case–control studies, with a publication year between 1966 and 2014 [9–17]. The additional search in July 2016 resulted in seven articles, of which three were selected based on their title and abstract. One of these three were excluded due to the presence of HIV infection and one was excluded due to low quality; only the remaining case report was included in this study [18], resulting in a total of ten included articles. The causality scores of the ten included studies are shown in Table 2. The total score per article ranged from 5 to 10, with two articles being allocated ten points [9, 17].

**Table 2:** Causality scores of the selected articles

Reference	Year of publication	Design	No. of cases	Age (years)	Strength	Consistency^a^	Specificity	Temporality	Gradient	Plausibility^a^	Coherence^a^	Experiment	Analogy^a^	Score/maximal score^b^
[9]	1966	Case series	5	4 to 12	NA	2	2	2	0	1	1	1	1	10/13
[10]	1978	Case report	1	16	NA	2	1	0	0	1	1	0	1	6/13
[11]	1980	Case report	1	28	NA	2	1	2	0	1	1	0	1	8/13
[12]	1982	Case–control	204	Avg. cases: 51.4 Avg. contr.: 53.2	0	2	1	0	0	1	1	0	1	6/15
[13]	1987	Case report	1	22	NA	2	1	2	0	1	1	1	1	9/13
[14]	2001	Case–control	45	Avg. cases: 43 Avg. controls: 52	1	2	1	0	0	1	1	0	1	7/15
[15]	2003	Case–control	150	Avg. cases: 37.8 Avg. contr.: 34.0	2	2	2	0	0	1	1	0	1	9/15
[16]	2007	Case–control	150	Avg. cases: 28.9 Avg. contr.: 27.6	0	2	2	0	0	1	1	0	1	7/15
[17]	2014	Case–control	1977^c^	Cases: 0 to 50+ Contr.: 0 to 50+	1	2	2	0	2	1	1	0	1	10/15
[18]	2016	Case report	1	15	NA	2	1	2	0	1	1	0	1	7/13

^a^These criteria were allocated scores for all articles simultaneously

^b^For case reports, the maximal score was 13, as the criterion of strength was not applicable

^c^After excluding HIV-infected patients

Four criteria were not applicable to individual articles but were scored simultaneously for all articles. For plausibility and analogy, all ten publications were allocated one point, as *T. gondii* is known to infect the brain and the development of epilepsy is, therefore, biologically plausible, while other parasitic infections (e.g. neurocysticercosis caused by *Taenia solium*) can cause epilepsy as well [19]. Along the same line, each article was allocated one point for coherence because the data didn’t seriously conflict with current knowledge. Regarding consistency, the studies comprised of various settings and different patient groups. Therefore, each study was allocated two points. Thus, all ten studies received the maximal score for these four characteristics; therefore, the minimal score was five points and the maximal score 15 (13 for case reports).

The strength of association was not applicable in the case reports or series. Of the five case–control studies, two reported a significant association between seropositivity for toxoplasmosis and epilepsy [15, 17], while it was not significant in the other three [12, 14, 16]. Yazar et al. found a significantly higher seropositivity rate in patients with cryptogenic epilepsy compared to patients with epilepsy due to a known cause or to healthy volunteers (*p* < 0.01) [15]. Kamuyu et al. performed a large case–control study in Africa and demonstrated a higher seropositivity rate for *T. gondii* in patients with epilepsy compared with age-matched community controls [odds ratio (OR) = 1.36; *p* < 0.015] [17]. Akyol et al. found no significant difference in seropositivity for *T. gondii* in 100 patients with epilepsy compared to 50 healthy controls [16]. Critchley et al. did not report statistical data but concluded that the prevalence of *T. gondii* antibodies in 204 patients with epilepsy was not in excess of that found in a non-epileptic population from another study [12]. Stommel et al. showed, after adjustment for age and gender, no difference in seropositivity between 22 patients with cryptogenic epilepsy and 23 healthy controls, but did report a higher antibody titre for *T. gondii* in patients with epilepsy compared with non-epileptic controls (Wilcoxon rank-sum test, *p* = 0.013) [14].

A gradient, here defined as higher *T. gondii* antibody titres among a group of patients with epilepsy, was found in one case–control study, in which high antibody titres were more strongly associated with epilepsy (OR = 1.36; *p* < 0.015) than lower antibody titres (OR = 1.25; *p* < 0.069) [17].

The case–control studies were more or less representative for a general conclusion (specificity), while single case reports were allocated one point.

Regarding temporality, only three case reports and the case series demonstrated unequivocally that the epileptic seizures took place after infection with *T. gondii* [9, 11, 13, 18].

Regarding the criterion of experiment, four individual patients had received anti-toxoplasmosis therapy. In four out of six patients, the epileptic insults resolved [9, 13], while two others did not recover [10, 18]. Due to lack of controls, the effect of treatment could not be interpreted with certainty. Only the case series and case report were allocated one point for this criterion.

In all, the strongest evidence for a causal relation was provided by one case report, one case series and one case–control study, each with a causality score of 9 or 10 points [9, 13, 17]. The remaining studies had a median causality score of 7 (range 5–9).

We found no selection bias, but six out of ten studies contained potential confounders, as indicated in Table 3 (in five, it was unsure whether the infection was pre- or postnatally acquired; in one study, immunodeficiency was not specifically excluded). The scientific impact of the selected articles was limited. The impact factor of the journals in which the selected studies were published varied from 1.1 to 4.5 and the number of citations per article varied from 0 to 31.

**Table 3:** Bias and confounding of selected articles

Reference	Bias and confounding			
	Information bias	Selection bias	Confounding	Total
[9]	0	0	0	0
[10]	0	0	1	1^a^
[11]	0	0	0	0
[12]	0	0	1	1^b^
[13]	0	0	0	0
[14]	0	0	1	1^b^
[15]	0	0	1	1^b^
[16]	0	0	1	1^b^
[17]	0	0	1	1^b^
[18]	0	0	0	0

^a^In this study, immunodeficiency was not specifically excluded

^b^In these five studies, it was unsure whether the infection was pre- or postnatally acquired

## Discussion

In this study, we performed a systematic assessment of causality of postnatal toxoplasmosis for the development of epilepsy in immune-competent patients. Worldwide, approximately 50 million individuals suffer from epilepsy. Among known causes are, e.g. genetic syndromes, prenatal or perinatal brain damage, infections of the brain or brain tumours, but in 60% of the patients the cause remains unknown [20]. It would, therefore, be relevant to know whether a common infection such as postnatal toxoplasmosis can cause epilepsy, and, if it can, how frequent this occurs.

Previous studies of epilepsy and toxoplasmosis included cases with congenital as well as with postnatal toxoplasmosis and, in addition, often included immunocompromised individuals. The recent meta-analysis by Ngoungou and colleagues of the relation between toxoplasmosis and epilepsy, which included studies of congenital toxoplasmosis and immunocompromised patients, found an estimated OR of 2.25 [95% confidence interval (CI) 1.15 to 3.93] [6]. In a large data analysis based on reported diagnoses from insurance records, a significant association between toxoplasmosis and epilepsy was reported (OR 3.51, 95% CI 3.00–4.12), thus in the same range as was found in the meta-analysis, but as a result of the study design, it could not be ascertained which proportion concerned patients with congenital toxoplasmosis or with an immune deficiency [1]. It was acknowledged that directionality and causality of observed relationships between toxoplasmosis and associated comorbidities were not clear. The specific focus of our study was to assess the causality of postnatal toxoplasmosis for the development of epilepsy specifically in immune-competent individuals.

Based on a literature search, ten articles were selected, five of which were case reports or case series and the remainder were case–control studies. In general, case reports are not used in formal meta-analyses, but for the particular purpose of this study, i.e. assessment of causality, the case reports actually provided the most convincing evidence by fulfilling the criterion for temporality, i.e. certainty that the exposure had occurred before and not after the patient had developed epilepsy. In all five case–control studies, it could not be excluded that infection with *T. gondii* and resulting positive serology had occurred after the patients had developed epilepsy because those studies included patients with previously diagnosed epilepsy and not de novo epilepsy as in the case reports. Even the presence of IgM antibodies, as was reported in some studies, does not prove recent infection because IgM may remain detectable for years [21]. Along the same line, because the time since infection was not known, a potential confounder in the case–control studies was that some or all of the patients could have had congenital toxoplasmosis which first manifested as epilepsy later in life. These limitations of the case–control studies justify the inclusion of case reports in the present assessment.

Acknowledging temporality as an essential causality factor, this was most convincingly illustrated in the case report by Beltrame and colleagues [18]. In summary, this report describes an immune-competent 15-year-old boy who developed epileptic insults after having eaten raw meat and vegetables while on a vacation in Ethiopia. Abnormalities were seen on the electroencephalogram (EEG) and serology for *T. gondii* was positive for both IgM and IgG with low avidity, together convincingly indicating recent infection. The patient received anti-toxoplasmosis therapy, after which the EEG pattern was restored to normal. However, 5 months later, the patient again had seizures with EEG abnormalities, and anti-epileptic drug treatment had to be restarted. In our opinion, this case history convincingly supports a causal relation between toxoplasmosis and epilepsy in an immune-competent person.

The presumed pathogenesis of how *T. gondii* would cause epilepsy is not yet fully understood, but if a causal relation actually exists, then the process is most likely multifactorial, with a contribution by both the immune response of the host and parasite-induced altered neurotransmission. The potential mechanisms contributing to the pathogenesis have recently been reviewed in detail by Ngoungou and colleagues [6]. In short, in the intermediate host, which includes humans, *T. gondii* forms cysts in several tissues, including the brain, infecting both neurons and glial cells [6]. A rupture of the cysts leads to an expulsion of bradyzoites, followed by a T cell immune response of the host, resulting in inflammation and scar tissue, which has been suggested as one of the main mechanisms leading to epilepsy [8, 18]. A recent study in mice showed that the presence of *T. gondii* tissue cysts led to an alteration in the gamma-aminobutyric acid (GABA) pathway, GABA being an inhibitory neurotransmitter [22]. This led to seizures in the infected mice and, while still highly speculative, this mechanism might contribute to the development of epilepsy in humans as well. In human patients with ocular toxoplasmosis, certain genotypes were overrepresented in immune-competent patients and, although speculative at present, strain-specific virulence, parasite stage and size or type of inoculum may contribute to the development of severe manifestations of toxoplasmosis in immune-competent individuals [23, 24].

Based on the present study, it is not possible to either finally prove or disprove a causal relation between postnatal toxoplasmosis and epilepsy. Because the seroprevalence of *T. gondii* is high and the proportion of patients with cryptogenic epilepsy is considerable, a chance co-occurrence could not be excluded. Not all of Hill’s criteria of causation have to be fulfilled to transform an association into belief of causation and Hill himself stated that our decision to take action is not a matter of causation but a matter of its merits [7]. Because epilepsy is a chronic disease that affects many aspects of a patient’s life and often requires prolonged use of anti-epileptic drugs, we think further study into a potentially treatable cause of epilepsy is justified. Obviously, experimental studies in humans are not possible for ethical reasons. A feasible study design could be to perform *T. gondii* serology, including IgM and IgG antibodies plus avidity in order to differentiate between recent and remote infection, in all incident cases of epilepsy. If available, a biobank of sera of de novo cases of epilepsy would provide suitable samples for such a study in a retrospective fashion. Patients with positive serology indicating recent toxoplasmosis could be included in a randomised controlled trial, comparing treatment with anti-toxoplasmosis therapy with placebo (no such study was found at http://www.clinicaltrials.gov). However, if a causal relation actually exists, the interval between toxoplasmosis and the first manifestation of epilepsy may vary and serology indicating past infection could still be relevant. Additional case–control studies including incident epilepsy cases and adequate controls would be useful. In this regard, it could be interesting to also study cell-mediated immune responses to *Toxoplasma*, which, after all, is mainly an intracellular pathogen, analogous to e.g. the interferon-gamma release assays as are presently used for the diagnosis of tuberculosis infection [25]. The balance between humoral and cellular responses might reveal a relevant association with neurological manifestations such as epilepsy.

A limitation of our study is the fact that the scoring system for the assessment of causality was arbitrary, but we think it, nevertheless, provides an objective, albeit possibly imprecise, measure of causality. The main limitation was the lack of high-quality data in the case–control studies because the temporal relation between infection with *T. gondii* and epilepsy could not be assessed. Finally, in some of the case reports, an underlying immune deficiency was not explicitly excluded, but because most of these reports were published long before the start of the HIV epidemic, it was unlikely that these patients were immunocompromised.

In conclusion, based on the available data, we think that postnatal toxoplasmosis in immune-competent individuals may cause encephalitis featuring as epilepsy, which can persist beyond the acute infection. More definitive proof of causality and an assessment of the frequency of this association require further study.

## Electronic supplementary material

Below is the link to the electronic supplementary material.

ESM 1 (DOC 41 kb)
