# Supplementary material for: A critical assessment of the association between postnatal toxoplasmosis and epilepsy in immune-competent patients
Source: Eur J Clin Microbiol Infect Dis. 2017 Jan 12;36(7):1111–7. doi: 10.1007/s10096-016-2897-0 (PMC5495839; doi:10.1007/s10096-016-2897-0)
Supplement: Supplementary file 1 — (DOC 41 kb) [file 10096_2016_2897_MOESM1_ESM.doc]

**SUPPLEMENT 1**

Search strategy with manuscript: **A critical assessment of the association between postnatal toxoplasmosis and epilepsy in immune-competent patients**

The search was performed on febr 5^th^ 2015 using below databases and search terms:

1. **PubMed**

<http://www.ncbi.nlm.nih.gov/entrez/query.fcgi?otool=leiden>

Search terms for 1:

("Toxoplasmosis"[Mesh] OR "Toxoplasma"[Mesh] OR toxoplasmos*[tw] OR "toxoplasma"[tw] OR toxoplasm*[tw] OR "t gondii"[tw] OR "t gondi"[tw] OR tgondi*[tw] OR "neurotoxoplasmosis"[tw] OR neurotoxoplasm*[tw]) AND ("Epilepsy"[Mesh] OR "epilepsy"[tw] OR epilep*[tw] OR "seizure"[tw] OR "seizures"[tw] OR seizur*[tw] OR convulsion*[tw]) NOT ("Toxoplasmosis, Congenital"[majr] OR "congenital toxoplasmosis"[ti])

1. **Embase**

<http://ovidsp.ovid.com/ovidweb.cgi?T=JS&PAGE=main&MODE=ovid&D=oemezd>

Search terms for 2:

("Toxoplasmosis"/ OR exp "Toxoplasma"/ OR toxoplasmos*.mp OR "toxoplasma".mp OR toxoplasm*.mp OR "t gondii".mp OR "t gondi".mp OR tgondi*.mp OR "neurotoxoplasmosis".mp OR neurotoxoplasm*.mp) AND (exp "seizure, epilepsy and convulsion"/ OR "epilepsy".mp OR epilep*.mp OR "seizure".mp OR "seizures".mp OR seizur*.mp OR convulsion*.mp) NOT (*"congenital toxoplasmosis"/ OR "congenital toxoplasmosis".ti) NOT conference abstract.pt

1. **Web of Science**

<http://isiknowledge.com/wos>

TS=(("Toxoplasmosis" OR "Toxoplasma" OR toxoplasmos* OR "toxoplasma" OR toxoplasm* OR "t gondii" OR "t gondi" OR tgondi* OR "neurotoxoplasmosis" OR neurotoxoplasm*) AND ("seizure, epilepsy and convulsion" OR "epilepsy" OR epilep* OR "seizure" OR "seizures" OR seizur* OR convulsion*)) NOT TI=("congenital toxoplasmosis")

NOT conference abstract.pt

1. **Cochrane**

<http://www.thecochranelibrary.com/view/0/index.html>

(("Toxoplasmosis" OR "Toxoplasma" OR toxoplasmos* OR "toxoplasma" OR toxoplasm* OR "t gondii" OR "t gondi" OR tgondi* OR "neurotoxoplasmosis" OR neurotoxoplasm*) AND ("seizure, epilepsy and convulsion" OR "epilepsy" OR epilep* OR "seizure" OR "seizures" OR seizur* OR convulsion*))

NOT TI=("congenital toxoplasmosis")
